# Integrative analysis of transcriptome and metabolism reveals potential roles of carbon fixation and photorespiratory metabolism in response to drought in Shanlan upland rice

**DOI:** 10.1186/s12864-022-09094-3

**Published:** 2022-12-30

**Authors:** Shubo Zhou, Lijing He, Wei Lin, Yi Su, Qing Liu, Mingnan Qu, Langtao Xiao

**Affiliations:** 1 Hunan Provincial Key Laboratory of Phytohormones and Growth Development, Hunan Agricultural University, Changsha, 410125 Hunan China; 2 Department of Agriculture and Forestry, Hainan Tropical Ocean University, Sanya, 572022 China; 3 College of fisheries and life science, Hainan Tropical Ocean University, Sanya, 572022 China; 4 Hainan Yazhou Bay Seed Laboratory, Sanya, 572025 China

**Keywords:** Transcriptomes, Metabolism, Photorespiration, Drought responsive genes, Shanlan upland rice

## Abstract

Shanlan upland rice is an important landrace rice resource and is characterized with high drought stress (DS) tolerance relative to cultivated rice. However, the molecular mechanism of DS response in Shanlan upland rice remains unclear. In this study, we performed an integrated analysis of transcriptome and targeted metabolism to decipher the key biological pathways that responded to drought tolerance using two Shanlan upland rice lines. Results show that SL10 possesses 64% higher photosynthetic efficiency (Pn) and 2-fold higher water use efficiency (WUE) than that in SL1 exposed to DS. The decrease in Pn by DS is not due to stomatal limitation effects for SL1. Transcriptome analysis suggests photosynthesis relevant pathways (photosynthesis-antenna proteins and carbon fixation) and photorespiration relevant pathway (glycine, serine and threonine metabolism) in SL1 under DS were significantly enriched in the down-regulated and up-regulated DEGs list, respectively. There are 412 up-regulated and 233 down-regulated drought responsive genes (DRGs) in SL10 relative to SL1 induced by DS. Targeted metabolism results suggest that the contents across five metabolites related to carbon fixation pathway were declined by 36 and 8% in SL1 and SL10 caused by DS, respectively. We finally summarized the both gene expression and metabolites involved in photorespiration and carbon fixation pathways in response to DS in both rice lines. This study provides valuable information for better understanding the molecular mechanism underlying drought tolerance in Shanlan rice.

**Supplementary Information:**

The online version contains supplementary material available at 10.1186/s12864-022-09094-3.

## Introduction

Rice (*Oryza sativa* L.) is one of the most important crops, but it consumes about 1350 billion cubic meters of water annually worldwide [1]*.* Water for the agricultural use becomes increasingly scarce due to the climate change and rapid expansion of industrialization and urbanization [2]. By year 2025, the irrigated rice in 15–20 million hectares in Asia will suffer from water scarcity and subjected thus to a persistent drought scourge [3]. Hence, this dilemma brings out great challenges and difficulties to produce more irrigated rice per unit land for formers, with limited water. Therefore, it is urgent to breed new rice cultivars by using new germplasm with great yield potential relying on improved water use efficiency under drought stress (DS) [4].

Upland rice (*Oryza sativa* L.) represents a type of landrace rice that dramatically differs from irrigated rice and acclimates to both DS and normal conditions [5]. Shanlan upland rice has a long history of cultivation on Hainan Island, China, and the typical cultivation method is slash-and-burn [6]. After a long period of domestication, it has become a special type of upland rice, and it is suitable for cultivation on hillsides and is especially tolerant to DS [7]. The great DS resistance in Shanlan upland rice is always related to high plant height, scattered panicles, many grains, a high seed setting rate, a high 1000 grain weight, high per plant yields, and rich genetic diversity [7]. Therefore, Shanlan upland rice could provide important germplasm for drought breeding in cultivated rice.

Stomatal adjustment is a key indicator, and it was mainly affected by cellular ion homeostasis and transport [8, 9]. Liu and colleagues observed higher chlorophyll content and water use efficiency (WUE) in Shanlan upland rice than that in cultivated rice varieties at the seedling stage under DS [10]. Under dry farming conditions, WUE and the stability of plasma membrane of Shanlan upland rice are higher than those of cultivated rice [7]. Furthermore, Liu and colleagues cloned a *HKT2* gene in Shanlan upland rice and elucidated the molecular mechanism of Na^+^ and K^+^ transport [11] (Liu et al., 2013). However, the molecular mechanism underlying drought resistance in Shanlan upland rice is less reported.

Omic-analysis including transcriptome technique has been extensively used to uncover the molecular mechanism of drought-resistant genes in rice [12, 13]. For examples, using transcriptome analysis, it was reported that the genes related to the photosynthetic system acted a critical role in upland rice domestication in rice varieties with different DS resistance levels [14]. Using a japonica rice cultivar, Yoo and colleagues claimed that *osphyB* (Os03g0309200) negatively regulates the tolerance to DS by repressing the activity of ascorbate peroxidase and modulating the total leaf area and stomatal density [15]. Niu and colleagues identified some unknown genes with high expression under DS treatment in Shanlan upland rice lines using transcriptome analysis [7]. In addition, metabolites involved in the plant central metabolic network serve as a hub for regulating carbon and energy metabolism under various stress conditions [16]. However, how central metabolites incorporate with gene expression to stimulate the adaptive response under abiotic stress in Shanlan rice remains unknown.

In this study, we performed an integrative analysis of transcriptome and central metabolism to elucidate the regulatory mechanism of drought responsive genes incorporates with metabolites in Shanlan upland rice. In this regard, we selected 2 Shanlan rice lines with distinct response to DS from 18 Shanlan rice that widely grown in China’s Hainan Island. The physiological and morphological parameters were determined in the two Shanlan rice lines in response to DS. Bioinformatic analysis from transcriptome dataset reveals carbon fixation and some important biological pathways such as photosynthesis and response to stress involved in drought response in Shanlan rice. The expression levels of drought responsive genes were validated with qPCR. Plant central metabolism including Cavin cycle and TCA cycle were used to extend the knowledge of adaptive response in Shanlan upland rice in response to DS together with transcriptome. We finally emphasized that the balance maintenance of between photosynthetic carbon fixation and photorespiratory metabolism plays crucial roles in Shanlan drought resistance response.

## Materials and methods

### Materials and growth conditions

In this study, we first screened 18 Shanlan rice accessions collected by Department of Agriculture and Forestry, Hainan Tropical Ocean University and Hainan Laboratory of Biodiversity and Rice Germplasm Innovation, Hainan University (Table S1). The rice accessions were derived from four different geographic regions in Hainan province, China, i.e., Baoting, Hainan Academy of Agricultural Sciences, Wuzhishan and Changjiang.

### Drought treatments

Simulation of DS experiments by polyethylene glycol (PEG) was carried out in a growth chamber according to the approach as previously documented [17]. The growth chamber environment is: 12 h/12 h photoperiod, 67 ~ 70% air humidity, 400 ~ 500 μmol m^− 2^ s^− 1^ PPFD and 32/25 °C air temperature. The 18 Shanlan rice seeds were soaked for 24 hours, germinated at a constant temperature of 30 °C for 24 h, and sowed in a petri dish. After 3 weeks of germination, the seedlings were transplanted into a rice culture medium. After 4 weeks of germination, the seedlings were treated with 25% (w/v) PEG6000 for 12 hours, the degree of leaf wilting and biomass were recorded (Table S1), and two Shanlan upland rice lines with contrasting drought-resistant levels were selected. We hence used Shanlan 1 (SL1, relative sensitive to DS) and Shanlan 10 (SL10, relative tolerance to DS) for further experiments. For an example, the SL1 is selected based on the largest decrease in biomass together with highest degree of leaf rolling caused by DS effects.

To confirm the performance of two Shanlan upland rice (SL1 and SL10) in response to DS, we grew the plants in pots (50 cm × 40 cm × 30 cm) containing commercial peat soil (Pindstrup Substrate no. 4) in the growth chamber. Six plants for each rice line were sowed one pot. The plants were irrigated every 2 days. DS treatments were conducted at 40 days after emergence and then we stopped watering for 20 days. The soil water contents in drought field block were decreased by 40% relative to control.

### Agronomic measurements

Plant height and tiller number were recorded in two Shanlan upland rice lines following DS treatments. Dry shoot biomass per plant were determined by collecting the above-ground plants at end of drought treatment, and determined the weight by drying oven at 80 °C for 12 h until constant weight.

### Photosynthetic measurements

We used a portable photosynthesis measurement system, Licor 6400XT (LICOR Corp., USA) to measure photosynthetic rates (*A*), stomatal conductance (g_s_), and transpiration (*E*). Instinct water use efficiency (WUE) was calculated by *A*/g_s_ and the stomatal limitation value (Ls) was calculated using the formula: Ls = 1–Ci/Ca [18]. The flow rates were 400 mmol s^− 1^, CO_2_, light density and temperature were 400 ppm, 1500 μmolm^− 2^ s^− 1^ and 27 °C, respectively. Four biological replicates were conducted.

In addition, we measured photochemical efficiency by using Multi-Function Plant Efficiency Analyser (M-PEA, PP-Systems). The *F*_o_ (minimum fluorescence) and *F*_m_ (maximal fluorescence) of leaves in the dark for 20 min were measured. After actinic light and saturation pulse values were applied, leaf chlorophyll fluorescence parameters, *F*_v_/*F*_m_ (maximum efficiency of PSII photochemistry under dark-adaption), and non-photochemical quenching (NPQ) were calculated [19].

### 3,3′-Diamino benzidine (DAB) staining

For H_2_O_2_ detection, rice leave samples were stained in 1 mg/mL DAB (pH 3.8) overnight at 25 °C, followed by 3 h of washing in 100% ethanol to remove chlorophyll and then 10 min of boiling for destaining.

### mRNA extraction and library preparation

Total RNA was extracted using TRIzol reagent according to the manufacturer’s instructions (Invitrogen, Carlsbad, CA). RNA degradation and contamination was monitored on 1% agarose gels, and purity was checked using the Nano-Photometer spectrophotometer (IMPLEN, CA, USA). RNA integrity was assessed using the RNA Nano 6000 Assay Kit of the Agilent Bioanalyzer 2100 system (Agilent Technologies, CA, USA). A total amount of 1.5 μg RNA per sample was used as input material for the RNA sample preparations. Sequencing libraries were generated using NEB Next Ultra RNA Library Prep Kit for Illumina (NEB, USA) following manufacturer’s recommendations and index codes were added to attribute sequences to each sample. The clustering of the index-coded samples was performed on a Bot Cluster Generation System using HiSeq 4000 PE Cluster Kit (Illumina, USA) as reported previously [20]. After cluster generation, the library preparations were sequenced on an Illumina Hiseq 4000 platform and 150 bp paired-end reads were generated.

### Read mapping and differentially expressed analysis

The quality of RNA-seq data (fastq files) was assessed by FastQC software (http://www.bioinformatics.babraham.ac.uk/projects/fastqc/). The adaption and reads with low quality from raw RNA-seq reads were trimmed using trim_galore softare (http://www.bioinformatics.babraham.ac.uk/projects/trim_galore/; adaptor of read1: AGATCGGAAGAGCACACGTCTGAACTCCAGTCAC; adaptor of read2: AGATCGGAAGAGCGTCGTGTAGGGAAAGAGTGT). RNA-seq analysis was performed by the STAR [21] software (version 2.5.3a; http://github.com/alexdobin/ STAR) with the rice reference genome IRGSP-1.0 as well as a gene transfer format (GTF) file (downloaded from Ensembl Plants http://plants.ensembl.org/).

After generating the genome index, the clean RNA-seq reads were aligned by STAR [21] with ‘—quantMode GeneCounts’ option to count number of reads per gene. Quantification of genes and isoforms was performed using cufflinks version 2.2.1. RNA-seq analysis was performed by the STAR [21] software (version 2.5.3a; http://github.com/alexdobin/STAR) with the rice reference genome IRGSP-1.0 as well as a gene transfer format (GTF) file (downloaded from Ensembl Plants http://plants.ensembl.org/). To identify differentially expressed genes (DEGs) and drought responsive gene (DRGs) between SL1 and SL10 under DS relative to control, a fragment per kilobase of transcript per million mapped reads (FPKM) method was applied to calculate the transcript abundance. Notably, DEGs were determined by the R package ‘DESeq2’ [22] with the reads counts reported by STAR [21]. Only genes with the adjusted *P*-value < 0.05 were considered as DEGs. To reduce transcription noise, each isoform/gene was included for analysis only if its FPKM values was > 0.01, a value was chosen based on gene coverage saturation analysis as described earlier [23].

### GO and KEGG analysis

For Gene Ontology (GO) annotation, we used an in-house Perl script UniProtKB GOA file (ftp.ebi.ac.uk/pub/databases/GO/goa). KOBAS (KEGG Orthology Based An-notation System, v2.0) was applied to identify reprogrammed biochemical pathways of each pathway as previously documented [24]. Both GO and KEGG terms with a corrected *P* value less than 0.05 were considered significantly enriched among the DEGs.

### Quantitative transcript measurements

Based on transcriptomes analysis, qPCR was used to confirm the expressed pattern of DRGs in response to DS in two Shanlan upland rice lines. The RNA sample used is same as transcriptome determinations. RNA extraction and reversed cDNA were performed as described previously [25]. The qPCR analysis was performed using SYBR Green PCR Master Mix (Applied Biosystems, Forster City, CA, USA, 4309155) and a real-time PCR system (ABI StepOnePlus, Applied Biosystems, USA). Primers for qPCR were designed using Primer Prime Plus 5 Software Version 3.0 (Applied Biosystems, USA). Primers were listed in Table S2. The qPCR running program consists of a reverse transcription step at 48 °C for 30 min and a Taq polymerase activation step at 95 °C for 30 s, followed by PCR: 45 cycles at 95 °C for 15 s, 61 °C for 20 s, and 72 °C for 30 s, ensued by a melting cycle. Assays were performed with three biological samples from each treatment, and measurements were replicated three times. The *actin1* gene was used as an expression control (housekeeping gene). Relative expression of a gene against *Actin1* was calculated as 2^−ΔΔCT^ (ΔCT = CT, gene of interest^−CT^), as described earlier [26]. The expression levels of three known drought-resistant genes OsLEA3–2 (LOC4332688) [27] and RePRP2.2 (LOC4343033) [28] were also tested as control.

### Metabolism determinations

Samples for metabolites measurements were collected immediately after finishing DS treatments for both SL1 and SL10 rice lines. Same marked section of leaves as gas exchange measurements were sampled. A targeted metabolic profiling in the leaves of both Shanlan upland rice lines (SL1 and SL10) collected in control and DS was prepared based on the LC-MS/MS (Triple Quad 6500, SCIEX) procedure as earlier described by [25]. Shortly, ~ 2.5 mg leaf samples collected from SL1 and SL10 Shanlan upland rice lines exposed by DS mentioned above were sampled in 2 ml Eppendorf tube containing pre-cooled metal beads, then immediately stored in liquid nitrogen. The samples were firstly extracted with ball mill at 30 Hz for 5 min, and then the extracted powder was dissolved in 1.5 ml methanol/chloroform mixture and incubated subsequently at − 20 °C for 5 h. Thereafter, the mixture was centrifuged at 2000 g and 4 °C for 10 min and eventually filtered with 0.43 μm organic phase medium (GE Healthcare, 6789–0404).

The metabolomic analysis was performed using metabolon software (Durham, NC, USA). The sample components were identified by comparing the retention time and mass spectra with those of the reference metabolites steeply. Regarding the metabolic compounds identification of each sample, it is highly recommended to consider the mass spectra with the entries of the mass spectra libraries NIST02 and the Golm metabolome database (http://csbdb.mpimp-golm.mpg.de/csbdb/gmd/gmd.html).

## Results

**Fig. 1:**
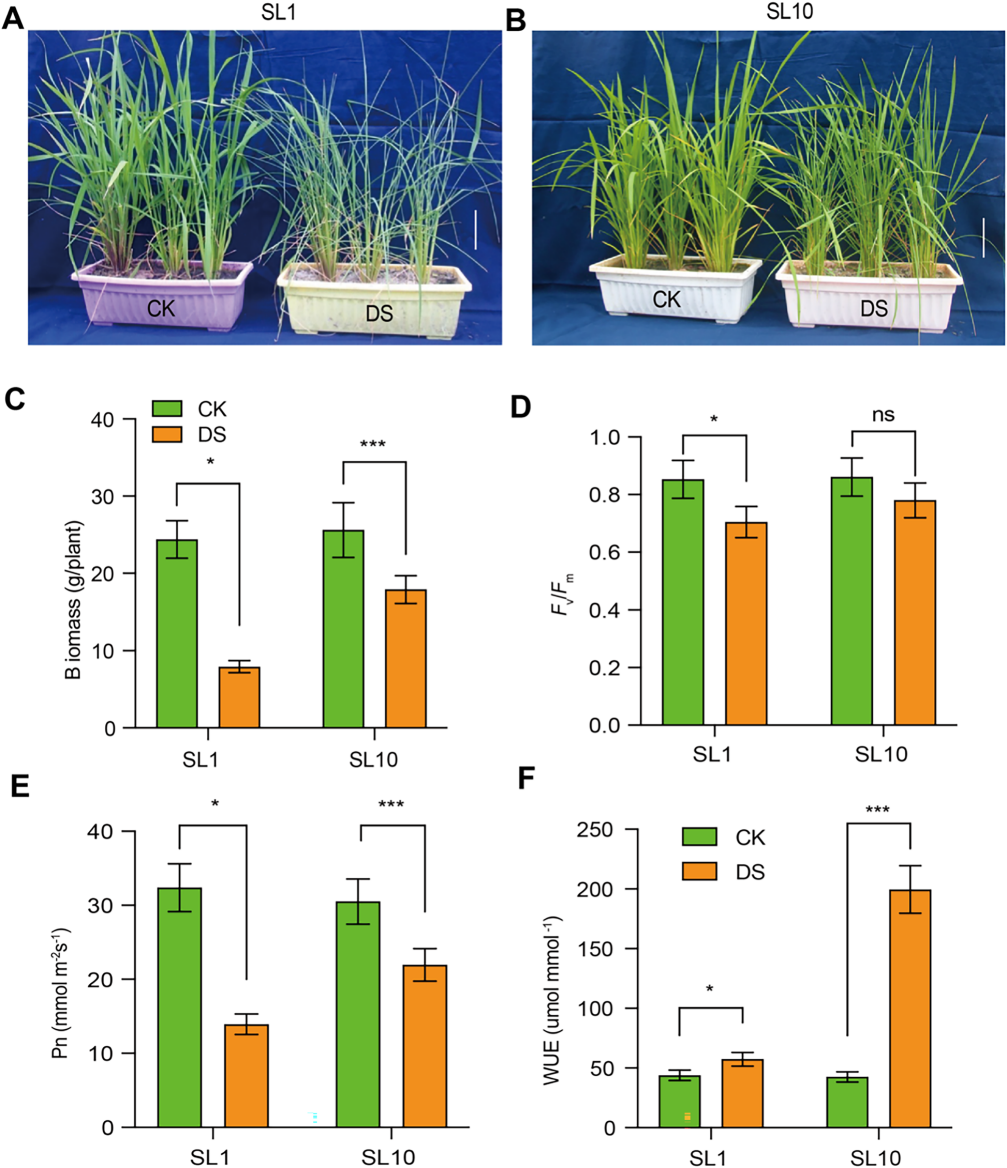
Morphology and physiology in response to drought treatments in two Shanlan rice lines. **A-B** images of two drought treated Shanlan rice (SL1 and SL10) for 12 days. Vertical white bar represents 10 cm scale. **C** biomass in two rice lines exposed to the drought stress (DS) treatment. **D-F** maximum photochemical efficiency (*F*_v_/*F*_m_), photosynthesis rates (Pn) and intrinsic water use efficiency (WUE) in two Shanlan rice lines exposed to the DS treatment. Symbols “*”, “**”, and “***” stand for the significance at *P* value < 0.05, 0.01, and 0.001 based on student t-test, respectively *n* = 6

In this study, we selected two Shanlan rice lines (SL1 and SL10) with contrasting drought tolerance from 18 Shanlan rice lines for pot-grown drought stress experiments (Table S2). Results show that the performance of SL1 was dramatically inhibited when exposed to 12 days DS treatment, while the performance of SL10 were slightly affected by DS (Fig. 1A-B). Consistently, the biomass decreased by 64% in SL1 while only 20% decrease in biomass in SL10 when exposed to 12 d DS treatment (Fig. 1C). Dramatic reduction in both *F*_v_/*F*_m_, Pn, g_s_, and E were observed in both Shanlan rice lines by DS treatment (Fig. 1D-E; Fig. S1A-B), while Ls, WUE and NPQ were slightly or significantly increased by DS treatment in both Shanlan rice lines (Fig. 1F; Fig. S1C-D).

There are 4 and 50% reduction in SL10 for *F*_v_/*F*_m_ and Pn, respectively, caused by DS, while there are 33 and 170% reduction in SL1 for the two parameters correspondingly (Fig. 1D-E; Fig. S1A-C), suggesting more reduction in photosynthetic capacity in SL1 than that in SL10. Consistently, we also observed higher WUE in SL10 than that in SL1 under DS treatment, suggesting better drought tolerance in SL1 than SL10. Furthermore, Ls was increased by 50% in SL10 while only 4% increase in SL1, suggesting that the reduction in Pn was not due to stomatal limitation in SL1. In addition, the higher increase in NPQ in SL1 under DS condition than that in SL10 suggests that more energy was dissipated by NPQ (Fig. S1D).

The activities of enzyme related to antioxidant system, including CAT, MDA, POD and SOD were slightly or dramatically enhanced by DS, while SL10 exerts higher increase in the activities of three enzymes than SL1 under DS (Fig. S2A-D). ROS are often associated with necrotic spot formation. We observed a higher level of H_2_O_2_ accumulation in SL1 line under DS condition, compared with that in SL10 (Fig. S3A-B).

To identify the drought-induced DEGs in both Shanlan rice lines, we performed a transcriptome analysis on the leaves in both SL1 and SL10, and found 4.3 M raw reads with 3.9 M clean reads. The percentage mapped reads in average were 95.6%, and the percentage of uniquely mapped clean reads accounting for total reads across samples was 96% across the 12 samples (Table S3). Q30, representing Phred value accounting for total bases was higher than 96%, and GC contents were around 52% across the 12 samples (Table S3). There are 95.3, 2.69 and 1.18% for the mapped reads to exon, intron and intergenic positions of genes, respectively (Fig. S4). The frequencies of both two nucleotides C and G were 7.4%, which is higher than that both A and T along with different the reads positions (Fig. S5A). The log_10_(FPKM) across 12 samples were 0.7 (Fig. S5B-C). There are 1524 up-regulated genes and 719 down-regulated genes between SL10 and SL1 under CK, while there are 243 up-regulated and 188 down-regulated genes between SL10 and SL1 under DS conditions (Fig. S5D).

**Fig. 2:**
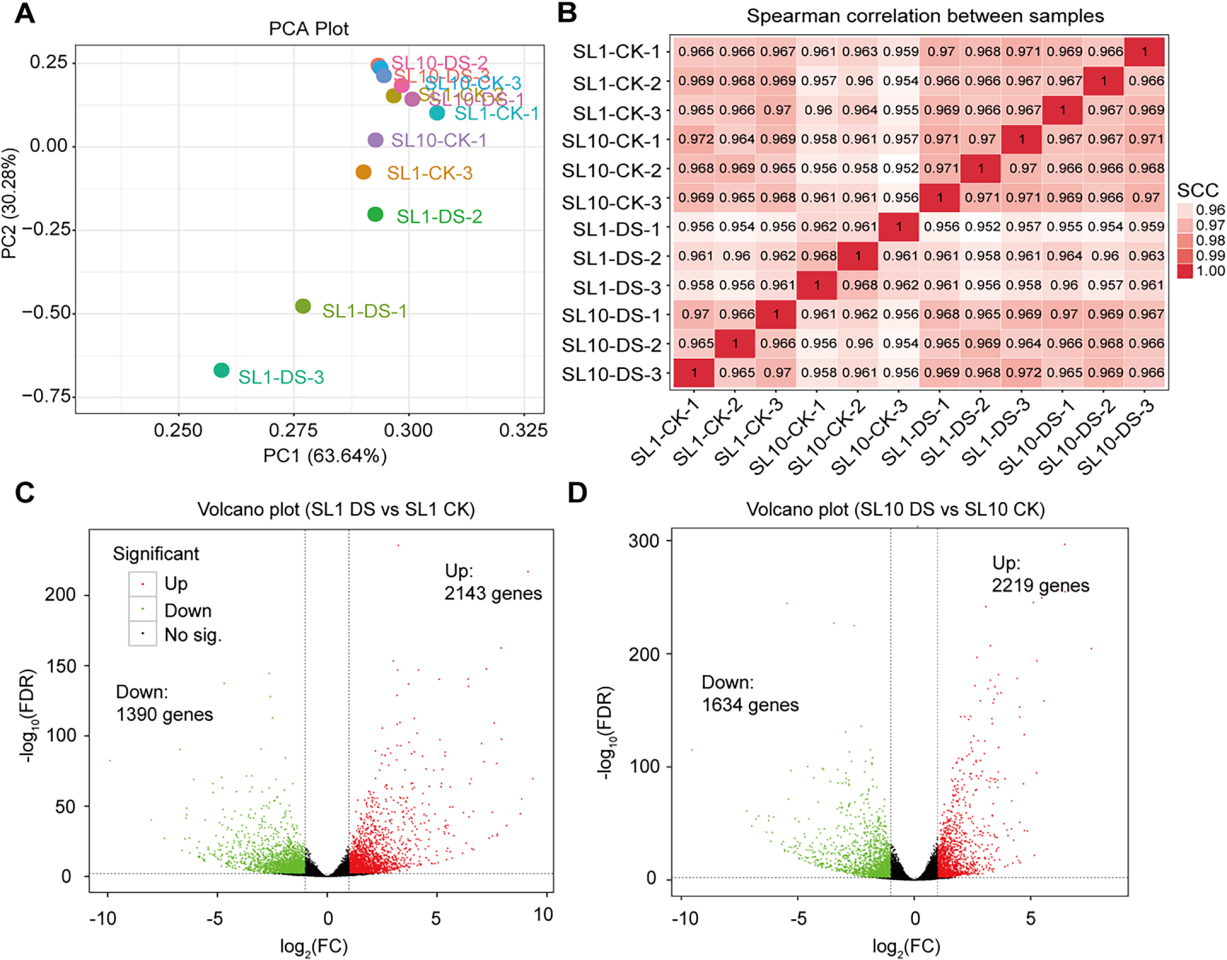
Principal component analysis on transcriptome analysis in two Shanlan rice lines exposed to drought stress treatment. **A** Principal component analysis (PCA) on the global transcripts. **B** Spearman correlation between samples. **C-D** volcano plot representing the differentially expressed genes between two Shanlan rice, i.e., SL1 (**C**) and SL10 (**D**) exposed to DS treatments

Relatedness of biological samples shows that strong correlation between SL1 and SL10 under CK condition, with spearman correlation coefficient > 0.97 (Fig. 2A-B). Consistently, principal component analysis on the abundance of 5208 genes shows that three biological samples of SL1-DS were distinctly separated from the other biological samples of SL1-CK, SL10-CK and SL10-DS. The accumulative score of PC1 and PC2 were 46 and 50% for both Shanlan rice lines under CK and DS, respectively (Fig. S6A-B). We identified 2143 up-regulated genes and 1390 down-regulated genes for SL1 exposed to DS treatment, while there are 2219 up-regulated and 1634 down-regulated genes for SL10 exposed to DS condition (Fig. 2C-D).

**Fig. 3:**
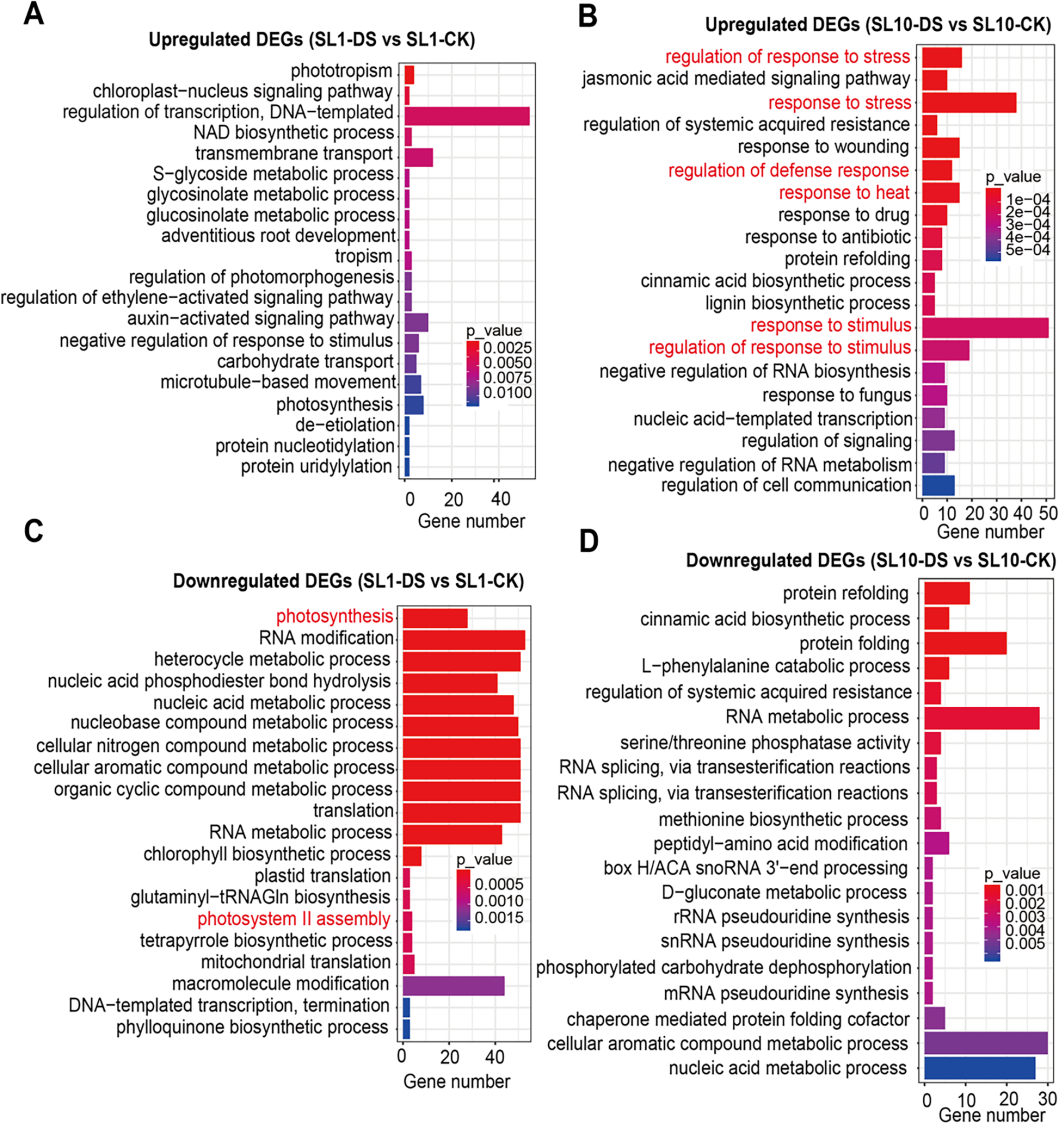
GO analysis on differentially expressed genes in two Shanlan rice lines exposed to either drought or control conditions. **A-B** GO analysis on up-regulated DEGs SL1 and SL10 in DS relative to CK. **C-D** GO analysis on down-regulated DEGs SL1 and SL10 in DS relative to CK. Pathways related to ROS signaling and carbon fixation were highlighted in red

**Fig. 4:**
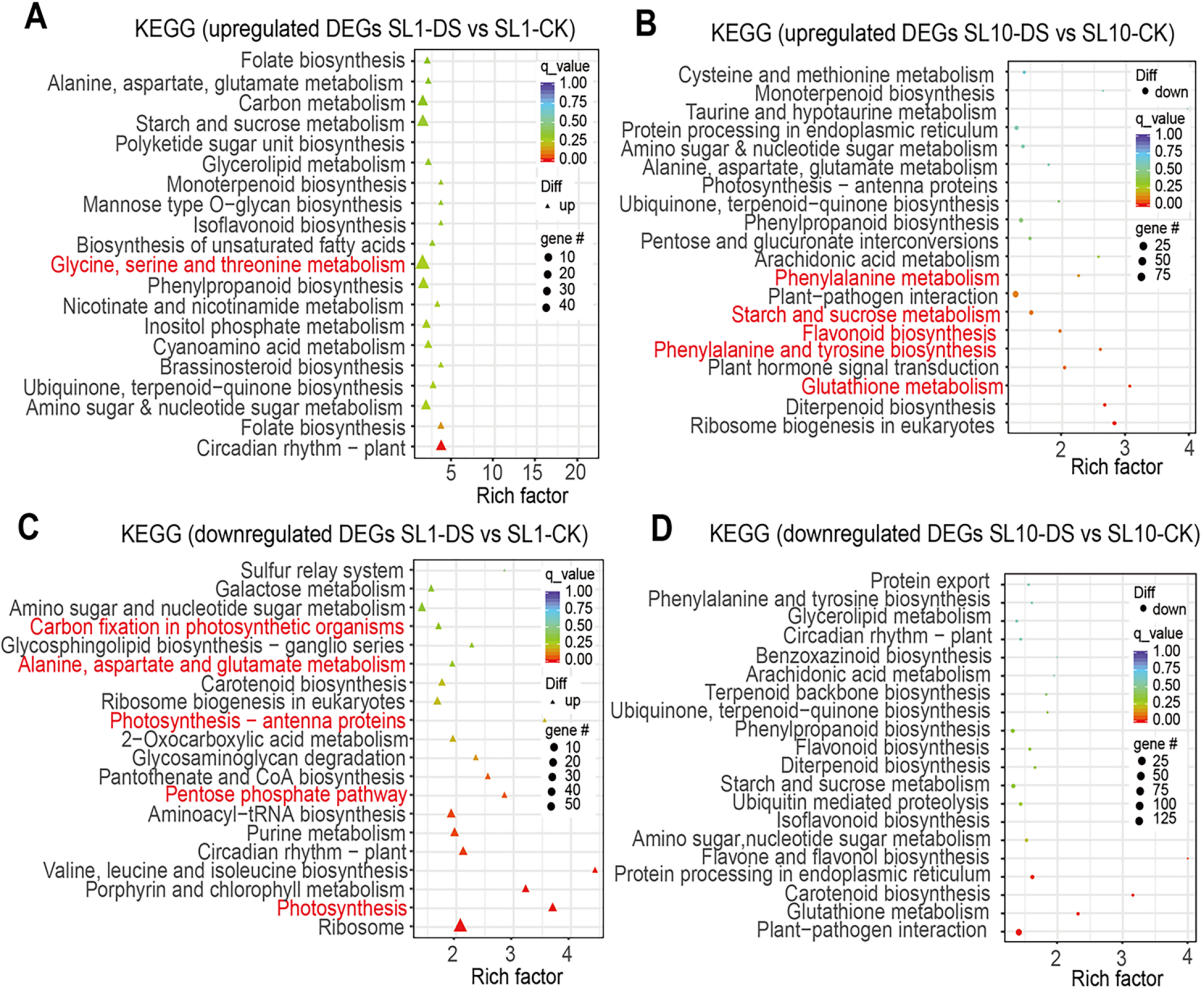
KEGG analysis on the DEGs in two Shanlan upland rice lines exposed to DS and control conditions. **A-B** KEGG analysis on up-regulated DEGs SL1 and SL10 in DS relative to CK. **C-D** KEGG analysis on down-regulated DEGs SL1 and SL10 in DS relative to CK. Pathways related to ROS signaling, photorespiration and carbon fixation were highlighted in red

GO analysis suggests that biological pathways of regulation of response to stress, regulation of defense response, response to stimulus and response to heat are significantly enriched in the list of up-regulated DEGs in SL10 exposed to DS (Fig. 3A-B). The analysis also shows that photosynthesis, photosystem II assembly and nitrogen compound metabolic process are significantly enriched in the list of down-regulated DEGs in SL1 exposed to DS, while it is different from SL10 exposed to DS (Fig. 3C-D). KEGG analysis reveals that metabolic pathways of phenylalanine metabolism, starch and sucrose metabolism, flavonoid biosynthesis, phenylalanine and tyrosine biosynthesis, glycine, serine and threonine metabolism are significantly enriched in the up-regulated DEGs list of SL10 induced by DS, while it is different from the metabolic pathways enriched in SL1 induced by DS (Fig. 4A-B). In terms of down-regulated DEGs in SL1 induced by DS, carbon fixation in photosynthetic organisms, alanine, aspartate and glutamate metabolism, glutathione metabolism, photosynthesis-antenna proteins, pentose phosphate pathways and photosynthesis were significantly enriched, while it is different from the metabolic pathways enriched in SL10 exposed to DS (Fig. 4C-D).

**Fig. 5:**
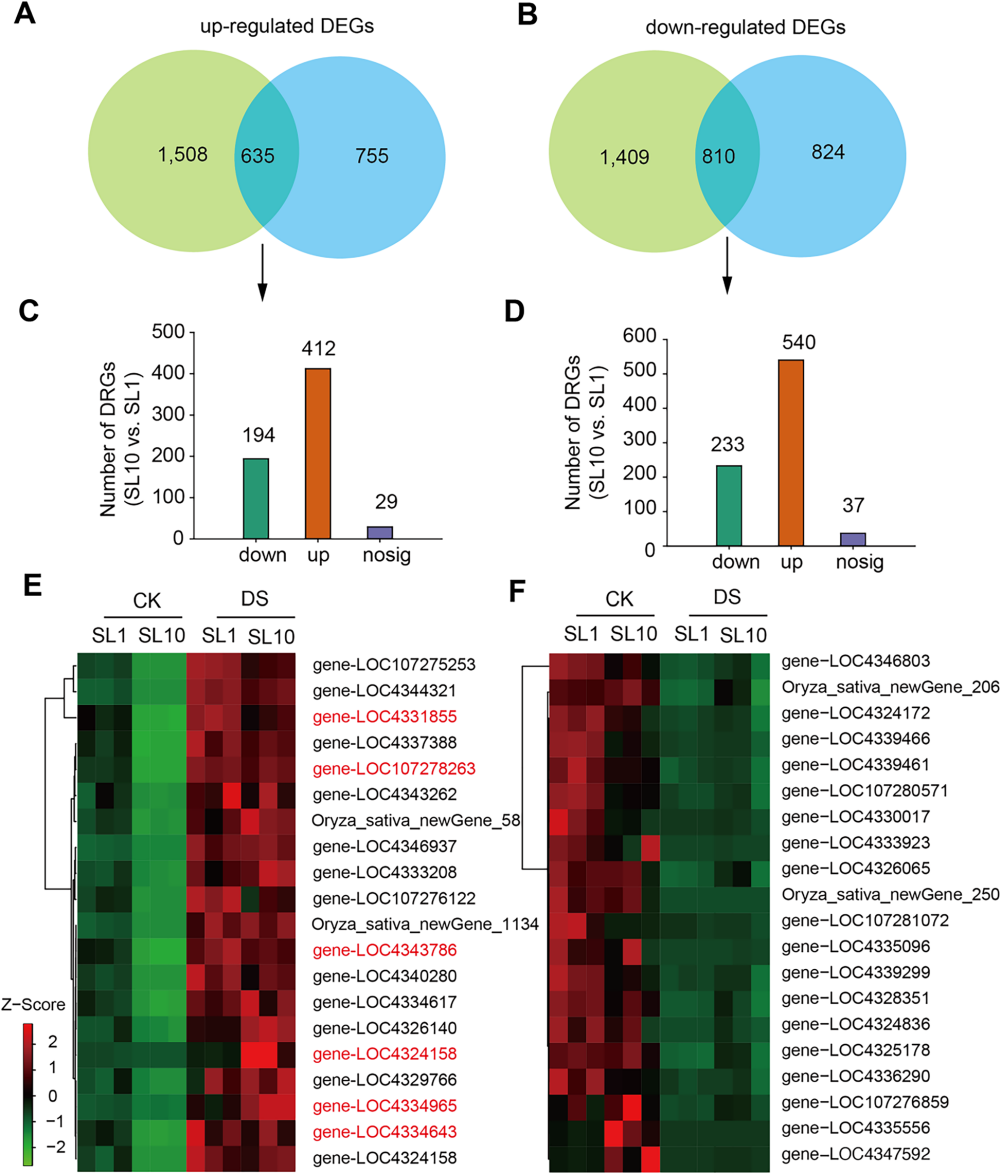
Drought responsive genes in two Shanlan upland rice lines. **A-B** venn diagram representing the up-regulated (**A**) and down-regulated (**B**) DEGs induced by drought stress for both SL1 and SL10. **C-D** numbers of DEGs in for the overlapped up-regulated (**C**) and down-regulated (**D**) DEGs gene list. **E-F** heatmap representing the relative abundance of genes in top 5% up-regulated (**E**) and down-regulated (**F**) DEG list. The detailed gene information in panels **E-F** were referred to Table S5 and S6, respectively. The expression levels of genes randomly selected were validated via qPCR as highlighted in red in panel **E**

To uncover the drought responsive genes (DRGs) in Shanlan upland rice lines, we compared the overlapped DEGs in both SL1 and SL10 induced by DS and found the 635 overlapped up-regulated DEGs. Herein, the DEGs with significant difference between SL10 and SL1 were considered as DRGs. Among them, there are 194 and 412 DRGs that were down-regulated and up-regulated, respectively, when compared them in SL10 with SL1 (Fig. 5A, C). In terms of overlapped down-regulated DEGs between SL10 and SL1 induced by DS, we found 810 DEGs. Among them, there are 233 and 540 down-regulated and up-regulated DRGs, respectively, when compared them in SL10 with SL1 (Fig. 5B, D). The relative abundance of top 20 DRGs were present in the 412 up-regulated DRGs and 233 down-regulated DRGs (Fig. 5E-F). To verify the reliability of the sequencing results, we selected the drought stress-related genes RAB21 and OsLEA3–2, and RePRP2.2, with defined functions as controls. Results show that the expression levels of the drought-related genes OsLEA3–2 and RePRP2.2 were significantly increased under DS treatment (Fig. S7). This means that our DS treatments for both SL1 and SL10 were sufficient. Furthermore, we conducted qPCR to validate the patterns of six gene expression in response to DS in the list of 20 up-regulated DRGs, including EP1 (LOC4331855), HSF1b (LOC4324158), EP2 (LOC4334965), EP3 (LOC107278263), EP4 (LOC4334643) and ZOS7 (LOC4343786) (Fig. S8).

**Fig. 6:**
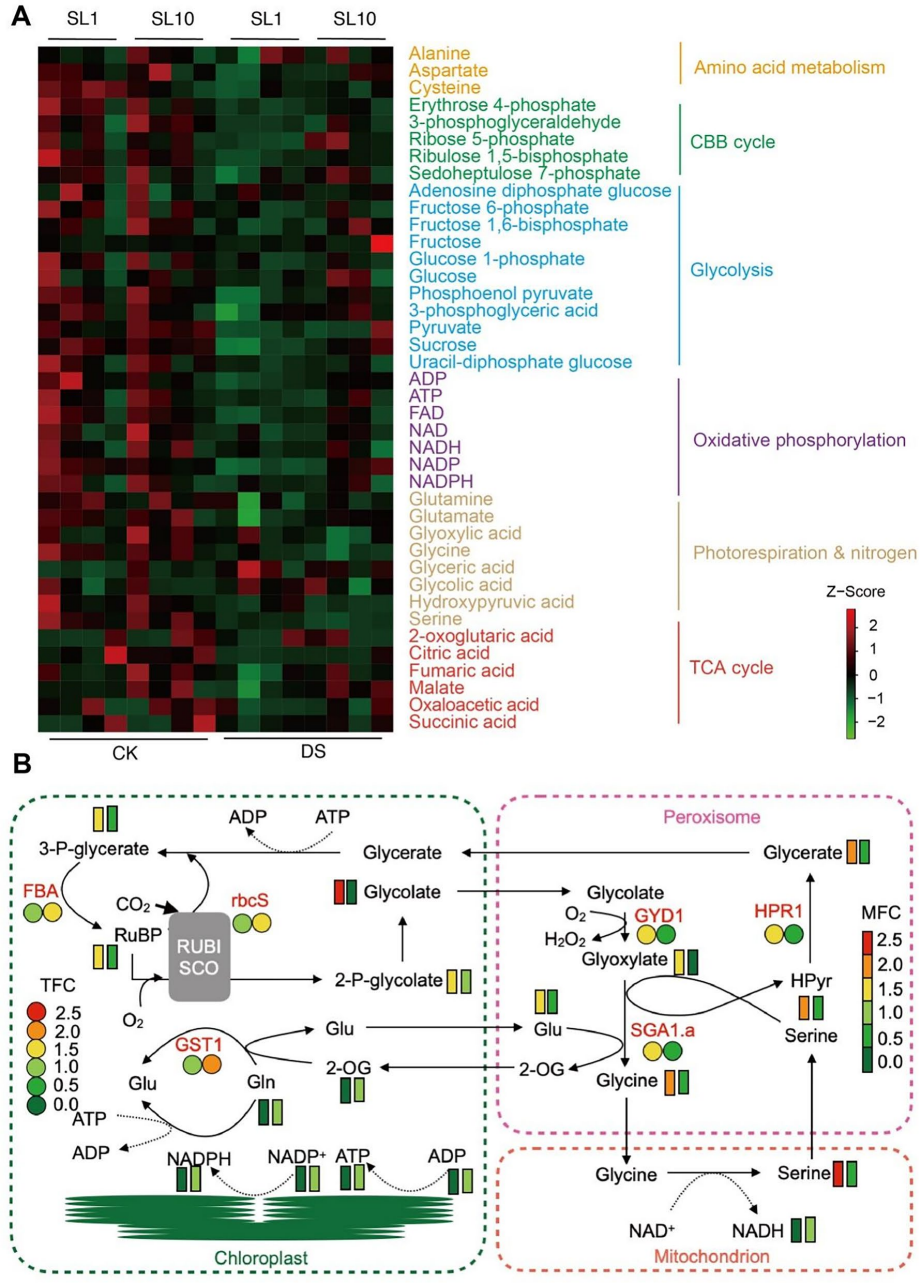
Changes of 40 metabolites in two Shanlan upland rice lines exposed to drought stress conditions. **A** heatmap representing the relative contents of metabolites related to photosynthetic process. The 40 metabolites are divided into six subgroups according to different metabolic pathways, i.e., amino acid metabolism, Calvin cycle (CBB cycle), glycolysis, oxidative phosphorylation, photorespiration & nitrogen metabolism and TCA cycle. **B** a summarized model showing the relative changes of metabolites and regulatory genes expression involved in photorespiration and antioxidant system. Figure 6B is not produced using KEGG database

Photorespiration coupled with CO_2_ assimilation is thought to act as a defense system against DS. Our transcriptome results suggest that the genes expression involved in photorespiration in SL1 were enhanced by up to 1.7-fold (Table 1), including SGA1.a (LOC4345962), DLDH1 (LOC4326980), GLYK (LOC4348728), GYD1 (LOC107277781) and HPR1 (LOC4327981), while the expression of gene involved in Calvin cycle were inhibited by up to 2.5-fold in SL1, suggesting DS induced the imbalance of carbon assimilation and photorespiration in SL1 but not SL10. To further elucidate the mechanism of photosynthetic process in response to DS in two Shanlan upland rice lines, we performed a targeted metabolism analysis on 40 metabolites related to amino acid metabolism, CBB cycle, glycolysis, oxidative phosphorylation, photorespiration & nitrogen and TCA cycle (Fig. 6A), and found that most pathways except for photorespiration pathway were dramatically enhanced in SL10 under DS compared it to SL1 under DS (Fig. 6A).

**Table 1:** Expression of genes involved in Calvin cycle and photorespiration pathway in two Shanlan upland rice lines (SL1 and SL10) under DS

Gene ID	log_2_FC (SL1 DS vs CK)	log_2_FC (SL10 DS vs CK)	Swiss-Prot_annotation
*Photorespiration*			
LOC4345962	1.22	0.08	SGA1.a, Serine-glyoxylate aminotransferase 1
LOC4326980	1.71	0.68	DLDH1, Dihydrolipoyl dehydrogenase 1
LOC4348728	1.36	0.19	GLYK, Phosphoglycerate kinase activity
LOC107277781	1.36	−0.25	GYD1, Glyoxylate reductase
LOC4327981	1.21	0.47	HPR1, Glycerate dehydrogenase HPR
*Carbon and nitrogen assimilation*			
LOC4341496	−1.91	1.06	FBA5, Fructose-bisphosphate aldolase 5
LOC4341770	−1.13	1.14	FBP1, Fructose-1,6-bisphosphatase
LOC4345685	−1.63	1.15	GAP3, Glyceraldehyde-3-phosphate dehydrogenase
LOC4351966	−2.28	−0.62	RBCS1, Ribulose bisphosphate carboxylase small chain
LOC4334316	−1.82	1.11	RPI1, Ribose-5-phosphate isomerase 4
LOC4327400	−1.67	−0.67	TPIC, Triosephosphate isomerase
LOC4333564	−2.56	−0.07	TRK1, Pyruvate dehydrogenase E1
LOC4330649	−1.39	−0.24	GST1, glutamine synthetase cytosolic isozyme 1

Collectively, we summarized a working model that representing the changes of photosynthetic carbon fixation and photorespiratory metabolism in response to DS in two Shanlan upland rice lines (SL1 and SL10) (Fig. 6B). DS induced the expression of genes related to Calvin cycle and nitrogen assimilation in SL10 (relatively tolerant to DS) but not SL1 (relatively sensitive to DS), such as FBA (LOC4341496), rbcS (LOC4351966) and GST1 (LOC4330649). Consistently, contents of metabolites related to the both pathways were pronounced including 3-P-glycerate, RuBP, and Gln. In contrast, DS inhibited the expression of genes related to photorespiratory pathway, such as SGA1.a (LOC4345962), GYD1 (LOC107277781) and HPR1 (LOC4327981).

## Discussions

### Photosynthetic decrease by DS is not due to stomatal limitation

Physiological regulation of drought response is related to stomatal adjustment, photosynthetic carbon assimilation, hormone regulation and some genetic factors [29]. There is a long-standing controversy as to whether drought limits photosynthetic CO_2_ assimilation through stomatal closure or by metabolic impairment in C_3_ plants [30]. Stomatal limitation to Pn (Ls) typically increases with an increase in water stress [31]. In current study, we found that a dramatic decrease in Pn by DS especially in drought relatively sensitive Shanlan rice lines (SL1), while the Ls was increased in DS in drought relatively tolerant Shanlan rice (SL10) but not in drought relatively sensitive Shanlan rice (SL1) under DS condition (Fig. S1C), suggesting that the natural variation in stomatal adjustment in regulation of photosynthetic adaption to DS [32, 33]. The phenomena reveals that plants experience severer DS leading to the damage of photosynthetic apparatus as observed in heat stress condition in maize [34], where RuBP regeneration and ATP synthesis are impaired [30]. This is in line with the greater reduction in the contents of ATP and R5P in SL1 than that in SL10 (Fig. 6A). We hence proposed that the reduction in Pn in SL1 might not be ascribe to stomatal limitation, but to some biochemical factors such as photosynthetically metabolic process involved in Calvin cycle.

### Photosynthetic assimilation coupling with photorespiration against DS

In addition to stomatal and ROS regulation to DS, photorespiration coupled with CO_2_ assimilation is also thought to act as a defense system against photoinhibition caused by DS in rice [35]. Photorespiration is a large and energy-consuming pathway that salvages byproducts of the reaction of Rubisco in the Calvin-Benson cycle [36]. The rates of energy consumption by photorespiration were reported to increase in response to DS [37]. In current study, we found some metabolites related to photorespiration pathway were accumulated by DS, such as glycolate, glyoxylate, glycine and serine in drought relatively sensitive Shanlan rice line (SL1), the increase is greater than that in SL10 (Fig. 6A). Consistently, the genes expression involved in photorespiration in SL1 were enhanced by up to 1.7-fold including GYD1 (LOC107277781), while the expression of gene involved in Calvin cycle including TRK1 (LOC4333564) were inhibited by up to 2.5-fold in SL1, suggesting DS induced the imbalance of carbon assimilation and photorespiration in SL1 but not SL10. In addition, it was also found that DS induced NPQ especially in SL1 but not SL10 (Fig. S1D), and that NPQ could also be stimulated in barley mutants with decreased activity of a photorespiratory enzyme, suggesting that photorespiration might consume excess light energy in Shanlan DS sensitive rice under DS [38].

### ROS scavenging system promotes drought resistance

When plants are exposed to stress, the generation of reactive oxygen species (ROS) is often one of the first responses [39]. Under DS conditions, the cellular metabolic process is interrupted, which inevitably drives ROS production [40]. Therefore, the avoidance of ROS production during DS is also an important strategy that enables plants to cope with limited-irrigation environments [40]. In this study, we found that in DS-resistance Shanlan upland rice (SL10), ROS scavenge relevant enzymes activities were dramatically pronounced such as catalase and superoxide dismutase (Fig. S2A,D). Consistently, many biological pathways related to the antioxidant system were up-regulated, such as glutathione metabolism, alanine, aspartate and glutamate metabolism (Fig. 4A). This supports that ROS signaling plays important roles in drought resistance in SL10 during DS treatments [41].

### Functions of C2H2 zinc finger proteins in DS response

Another important pathway for drought response is ABA signaling transduction. It is clear that ABA binds directly to the PYR/PYL family of ABA receptors, resulting in the inhibition of type 2C phosphatases (PP2C) and activation of downstream ABA signaling, hence inducing the expression of various DRGs, such as ABI1 and ABI2 [8, 42], and some transcription factors in rice including OsMADS25 [43], OsABF1 [44]. Some DRGs involved in ABA-dependent pathways were reported previously in Shanlan upland rice, such as AWPM-19-like (Os05g0381400) [7]. In current study, biological pathways related to response to stress were highly enriched in the list of up-regulated list DEGs in SL10 (Fig. 3C). In addition, the expression of these genes was induced under DS condition. In current study, we found a C2H2 zinc finger protein, ZOS7 (LOC4343786) gene to be highly expressed by DS especially in SL10 (Fig. 5E; Fig. S8F; Table S4). ZOS7, is an OsMYB60 binding partner and involved in leaf development in rice [45]. It was reported that some genes involved in C2H2 zinc finger proteins participate in ABA-mediated drought response [46]. The genetic and molecular mechanisms of ZOS7 involved in DS response are of great interest to be further elucidated in the future studies.

## Conclusions

Shanlan upland rice provides important germplasm towards drought breeding in cultivated rice. However, the molecular mechanism underlying photosynthetic adaptive response in Shanlan rice to DS remains unclear. In this study, we found that photosynthetic decrease by DS in SL1 is not due to stomatal limitation, but is related to increased photorespiration and impaired photosynthetic apparatus. SL10 possesses better antioxidant and ROS scavenge system, facilitating to high expression of some DRGs such as ZOS7. By integrative analysis of central metabolism and transcriptome, we proposed that the imbalance between photosynthetic carbon assimilation and photorespiration play critical role in drought response in Shanlan rice used in this case study.

## Supplementary Information

**Additional file 1: Table S1.** Drought resistence levels of 18 Shanlan upland rice lines collected from different regions of Hainan, China. **Table S2.** Primer list for qPCR test used in this study. **Table S3.** Statistical table of sequence alignment results between sample sequencing data and selected reference genomes. **Table S4.** Upreglated drought responsive genes in SL10 relative to SL1 under drought condition. **Table S5.** Downregulated drought responsive genes in SL10 relative to SL1 under drought condition. **Table S6.** Amounts of metabolites related to photosynethetic effiency in SL10 relative to SL1 under drought condition based on metabolic determinations.

**Additional file 2: Figure S1.** Physiological and photochemical parameters in response to drought treatments in two Shanlan upland rice lines. A-B stomatal conductance (g_s_) and transpiration rates (E). C-D stomatal limitation (Ls) and non-photochemical quenching (NPQ). *n* = 5 for panels A-B and *n* = 10 for panels C-D. Symbols “*”, “**”, and “***” stand for the significance at *P* value < 0.05, 0.01, and 0.001 based on student *t*-test, respectively. **Figure S2.** Activities of antioxidant enzymes in response to drought treatments in two Shanlan upland rice lines. A-D catalase (CAT), malondialdehyde (MDA), peroxidase (POD) and superoxide dismutase (SOD), respectively. Symbols “*”, “**”, and “***” stand for the significance at *P* value < 0.05, 0.01, and 0.001 based on student t-test, respectively. *n* = 3. **Figure S3.** Detection of H_2_O_2_ in two Shanlan rice lines exposed to drought condition. A leaf images taken before DAB staining. B DAB staining images. **Figure S4.** Statistical analysis on the gene structure in 12 biological samples of Shanlan upland rice based on transcriptome analysis. **Figure S5.** Statistical analysis on the read numbers in different Shanlan upland rice exposed to DS treatment. A percentage of based distribution in four nucleotides in two reads. B-C distribution of log_10_(FPKM) in different samples of Shanlan upland rice exposed to DS treatments. D differentially expressed genes in different samples. **Figure S6.** Principal component analysis (PCA) on the global transcripts in two Shanlan upland rice lines exposed to drought stress treatment. A SL1 and SL10 under CK. B SL1 and SL10 under DS. **Figure S7.** qPCR validation of known drought resistance genes induced by drought stress. A OsLEA3–2 (LOC4332688). B RePRP2.2 (LOC4343033). Leaf samples from SL1 and SL10 for each condition (either CK or DS) were pooled together for qPCR validation. *P* values in each comparison were determined based on student *t*-test, respectively. *n* = 3 (biological replicates). **Figure S8.** qPCR validation of DRGs in two Shanlan upland rice lines induced by drought stress. A-E EP1(LOC4331855), HSF1b(LOC4324158), EP2(LOC4334965), EP3(LOC107278263), EP4(LOC4334643) and ZOS7 (LOC4343786). *P* values in each comparison were determined based on student *t*-test, respectively. *n* = 3 (biological replicates).

## Data Availability

All data is available in the manuscript or the supplementary materials. All related sequencing data is deposited in NCBI Sequence Read Archive (SRA) database with the link of https://www.ncbi.nlm.nih.gov/sra?term= PRJNA793928. The bioProject accession is PRJNA793928.
