# Supplementary material for: Integrative analysis of transcriptome and metabolism reveals potential roles of carbon fixation and photorespiratory metabolism in response to drought in Shanlan upland rice
Source: BMC Genomics. 2022 Dec 30;23:862. doi: 10.1186/s12864-022-09094-3 (PMC9805275; doi:10.1186/s12864-022-09094-3)
Supplement: Supplementary file 2 — Additional file 2: Figure S1. Physiological and photochemical parameters in response to drought treatments in two Shanlan upland rice lines. A-B stomatal conductance (gs) and transpiration rates (E). C-D stomatal limitation (Ls) and non-photochemical quenching (NPQ). n = 5 for panels A-B and n = 10 for panels C-D. Symbols “*”, “**”, and “***” stand for the significance at P value < 0.05, 0.01, and 0.001 based on student t-test, respectively. Figure S2. Activities of antioxidant enzymes in response to drought treatments in two Shanlan upland rice lines. A-D catalase (CAT), malondialdehyde (MDA), peroxidase (POD) and superoxide dismutase (SOD), respectively. Symbols “*”, “**”, and “***” stand for the significance at P value < 0.05, 0.01, and 0.001 based on student t-test, respectively. n = 3. Figure S3. Detection of H2O2 in two Shanlan rice lines exposed to drought condition. A leaf images taken before DAB staining. B DAB staining images. Figure S4. Statistical analysis on the gene structure in 12 biological samples of Shanlan upland rice based on transcriptome analysis. Figure S5. Statistical analysis on the read numbers in different Shanlan upland rice exposed to DS treatment. A percentage of based distribution in four nucleotides in two reads. B-C distribution of log10(FPKM) in different samples of Shanlan upland rice exposed to DS treatments. D differentially expressed genes in different samples. Figure S6. Principal component analysis (PCA) on the global transcripts in two Shanlan upland rice lines exposed to drought stress treatment. A SL1 and SL10 under CK. B SL1 and SL10 under DS. Figure S7. qPCR validation of known drought resistance genes induced by drought stress. A OsLEA3–2 (LOC4332688). B RePRP2.2 (LOC4343033). Leaf samples from SL1 and SL10 for each condition (either CK or DS) were pooled together for qPCR validation. P values in each comparison were determined based on student t-test, respectively. n = 3 (biological replicates). Figure S8. qPCR validati [file 12864_2022_9094_MOESM2_ESM.docx]

**Supplementary Figures**


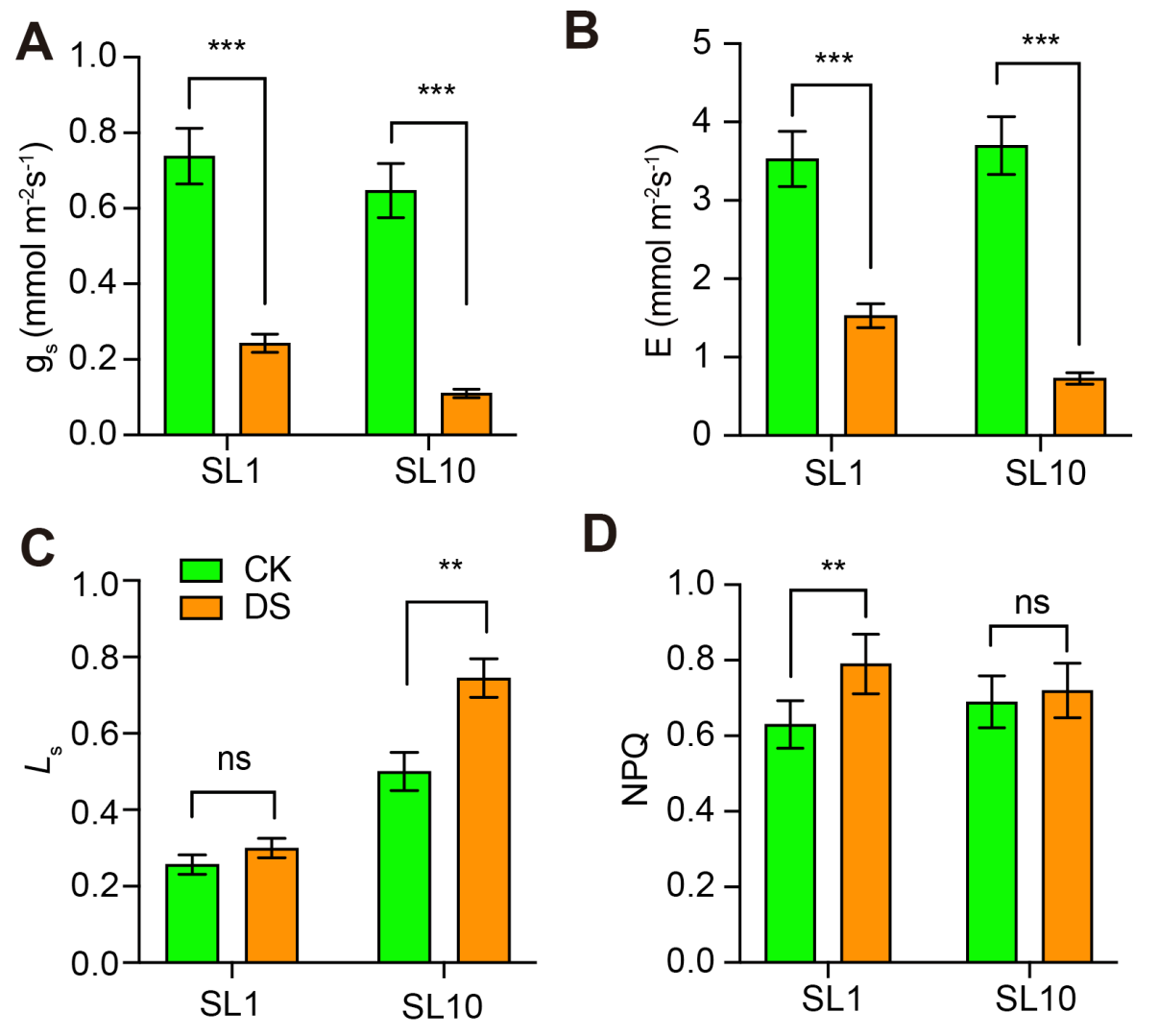


**Figure S1.** Physiological and photochemical parameters in response to drought treatments in two Shanlan upland rice lines. **A-B**, stomatal conductance (g_s_) and transpiration rates (E). **C-D**, stomatal limitation (Ls) and non-photochemical quenching (NPQ). *n*=5 for panels **A-B**, and *n*=10 for panels **C-D**. Symbols “*”, “**”, and “***” stand for the significance at *P* value <0.05, 0.01, and 0.001 based on student *t*-test, respectively.


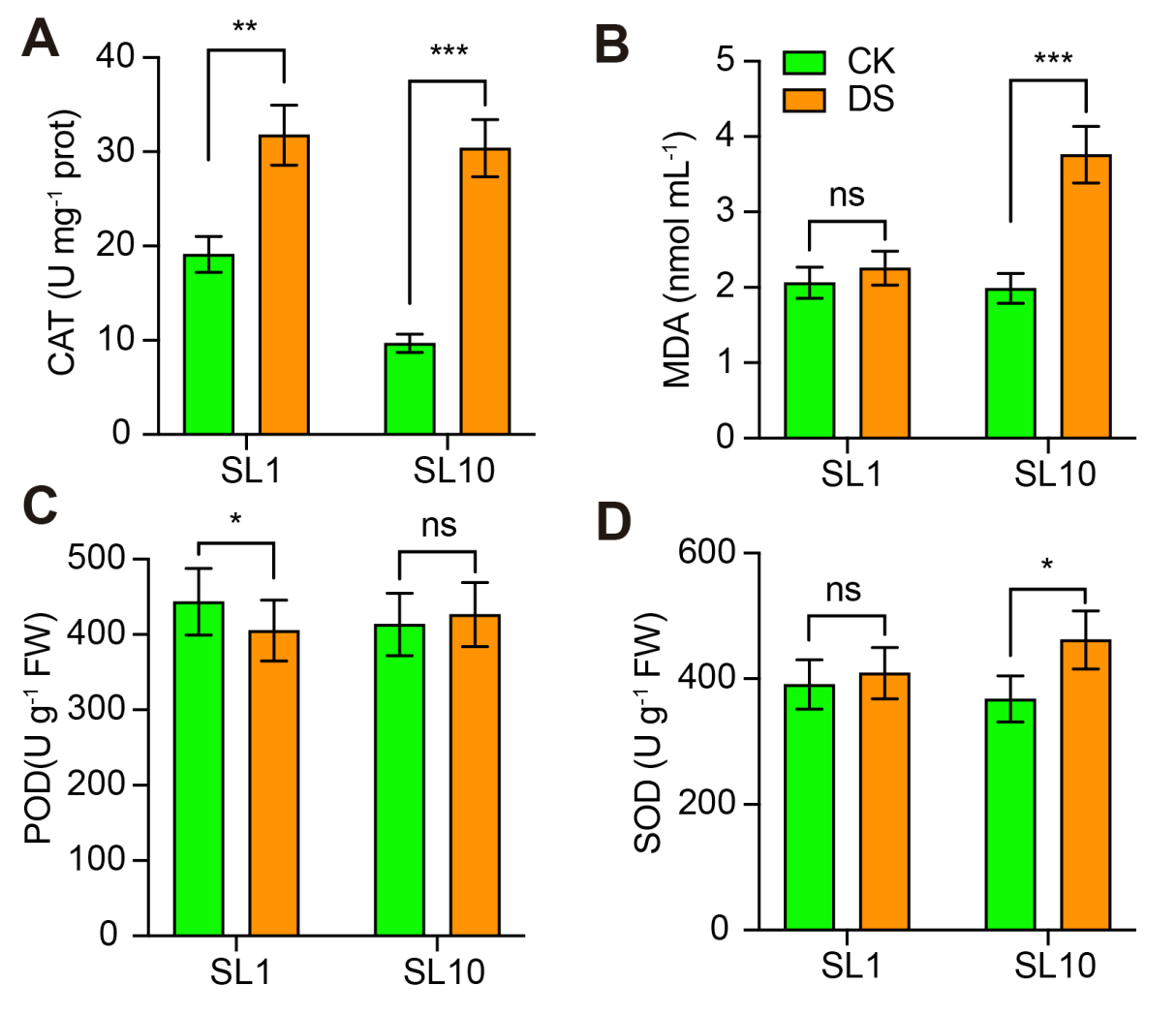


**Figure S2.** Activities of antioxidant enzymes in response to drought treatments in two Shanlan upland rice lines. **A-D**, catalase (CAT), malondialdehyde (MDA), peroxidase (POD) and superoxide dismutase (SOD), respectively. Symbols “*”, “**”, and “***” stand for the significance at *P* value <0.05, 0.01, and 0.001 based on student t-test, respectively. *n*=3.

**
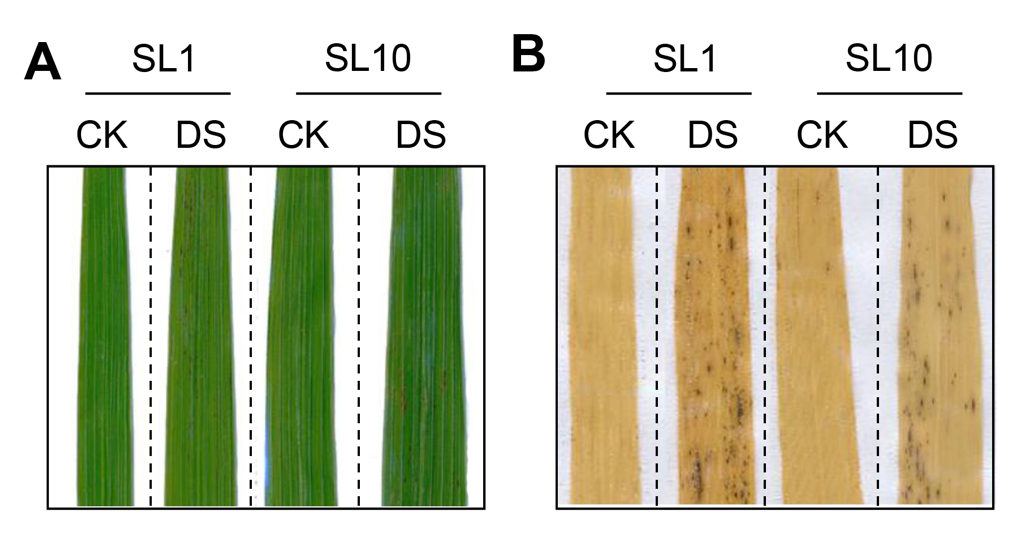
**

**Figure S3.** Detection of H_2_O_2_ in two Shanlan rice lines exposed to drought condition. **A**, leaf images taken before DAB staining. **B**, DAB staining images.


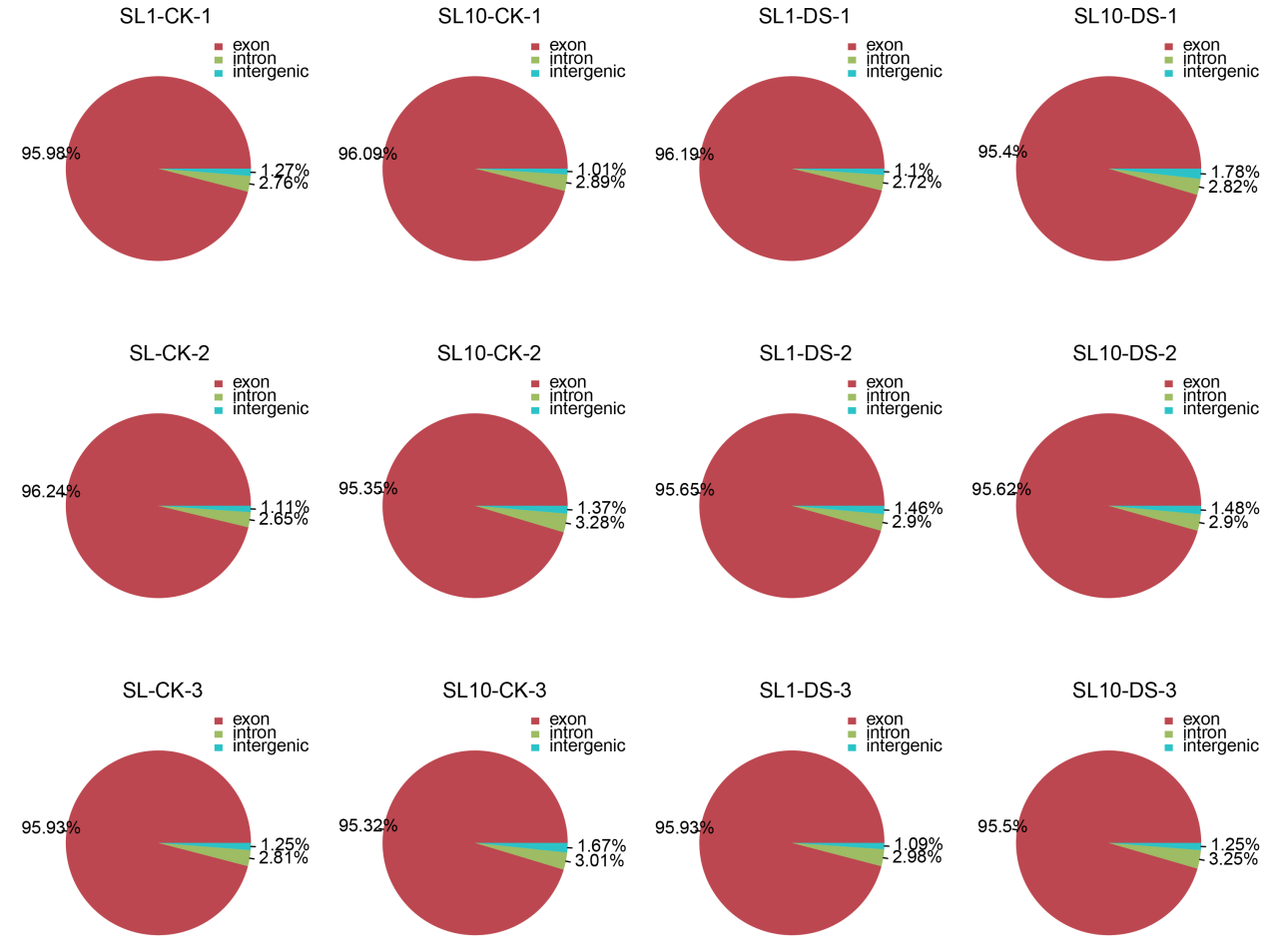


**Figure S4.** Statistical analysis on the gene structure in 12 biological samples of Shanlan upland rice based on transcriptome analysis.


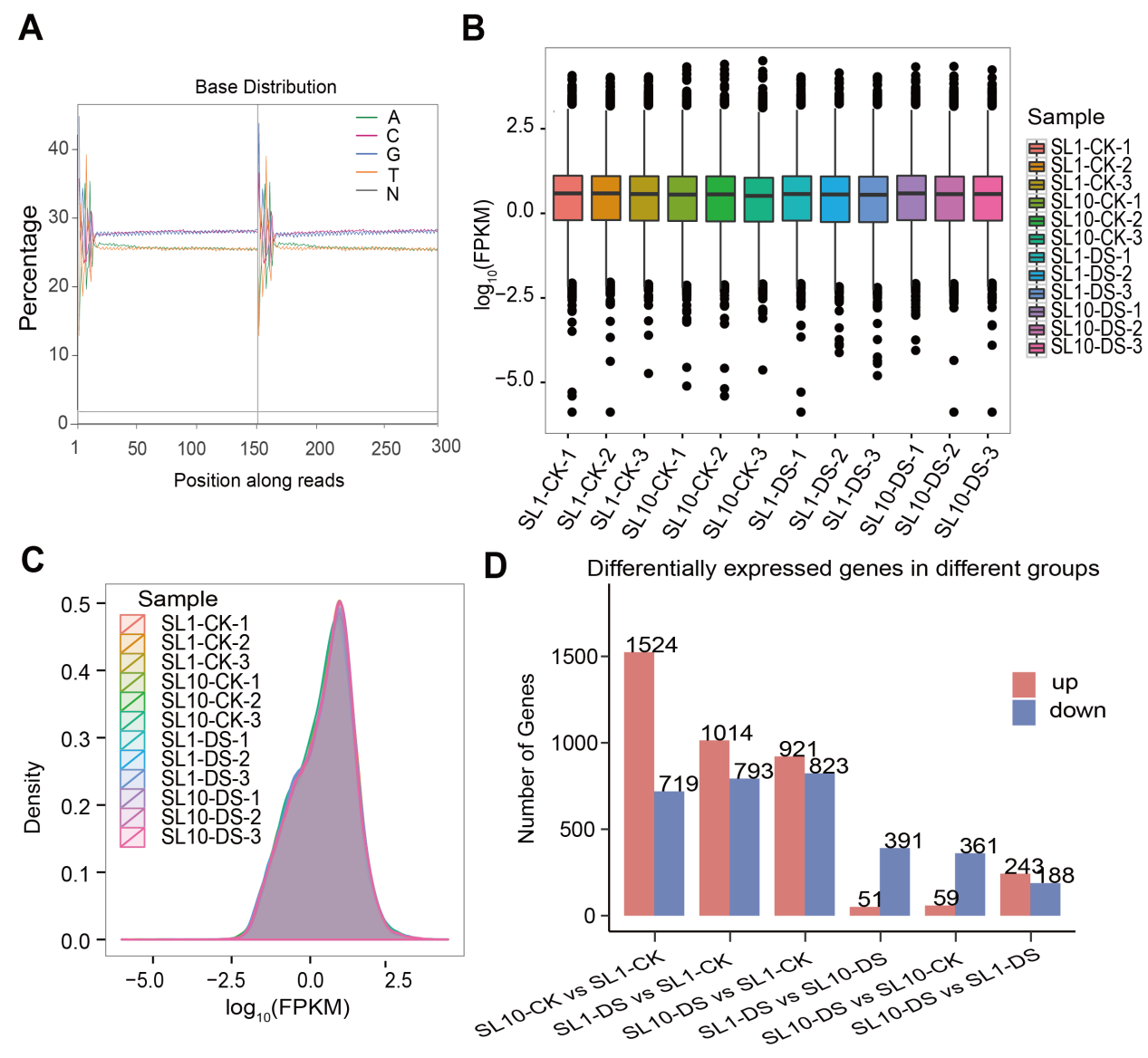


**Figure S5.** Statistical analysis on the read numbers in different Shanlan upland rice exposed to DS treatment. **A**, percentage of based distribution in four nucleotides in two reads. **B-C**, distribution of log_10_(FPKM) in different samples of Shanlan upland rice exposed to DS treatments. **D**, differentially expressed genes in different samples.


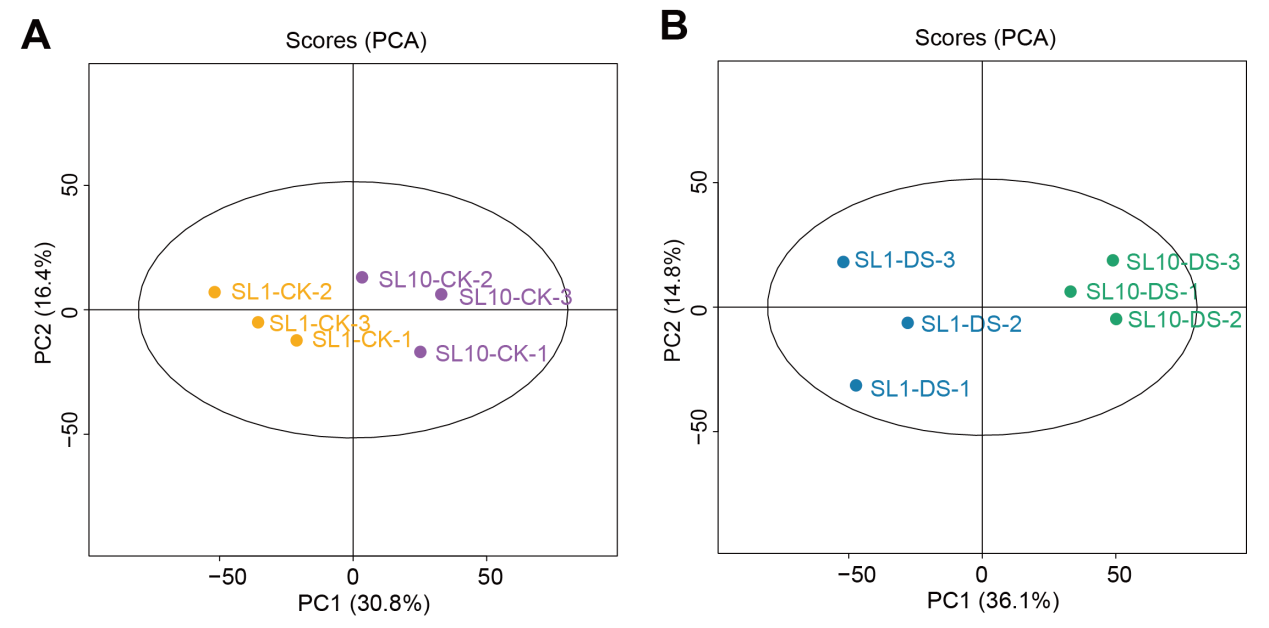


**Figure S6.** Principal component analysis (PCA) on the global transcripts in two Shanlan upland rice lines exposed to drought stress treatment. **A**, SL1 and SL10 under CK. **B**, SL1 and SL10 under DS.


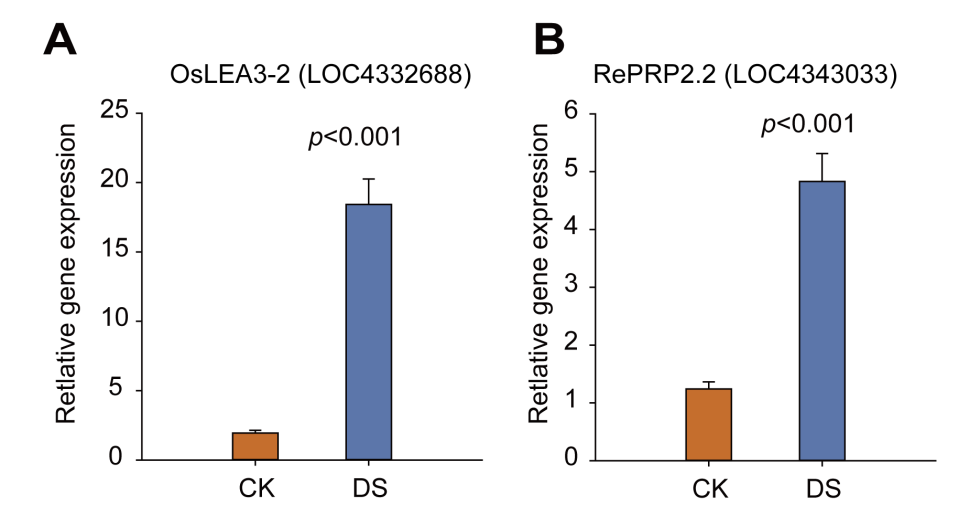


**Figure S7.** qPCR validation of known drought resistance genes induced by drought stress. **A**, OsLEA3-2 (LOC4332688). **B**, RePRP2.2 (LOC4343033). Leaf samples from SL1 and SL10 for each condition (either CK or DS) were pooled together for qPCR validation. *P* values in each comparison were determined based on student *t*-test, respectively. *n*=3 (biological replicates).


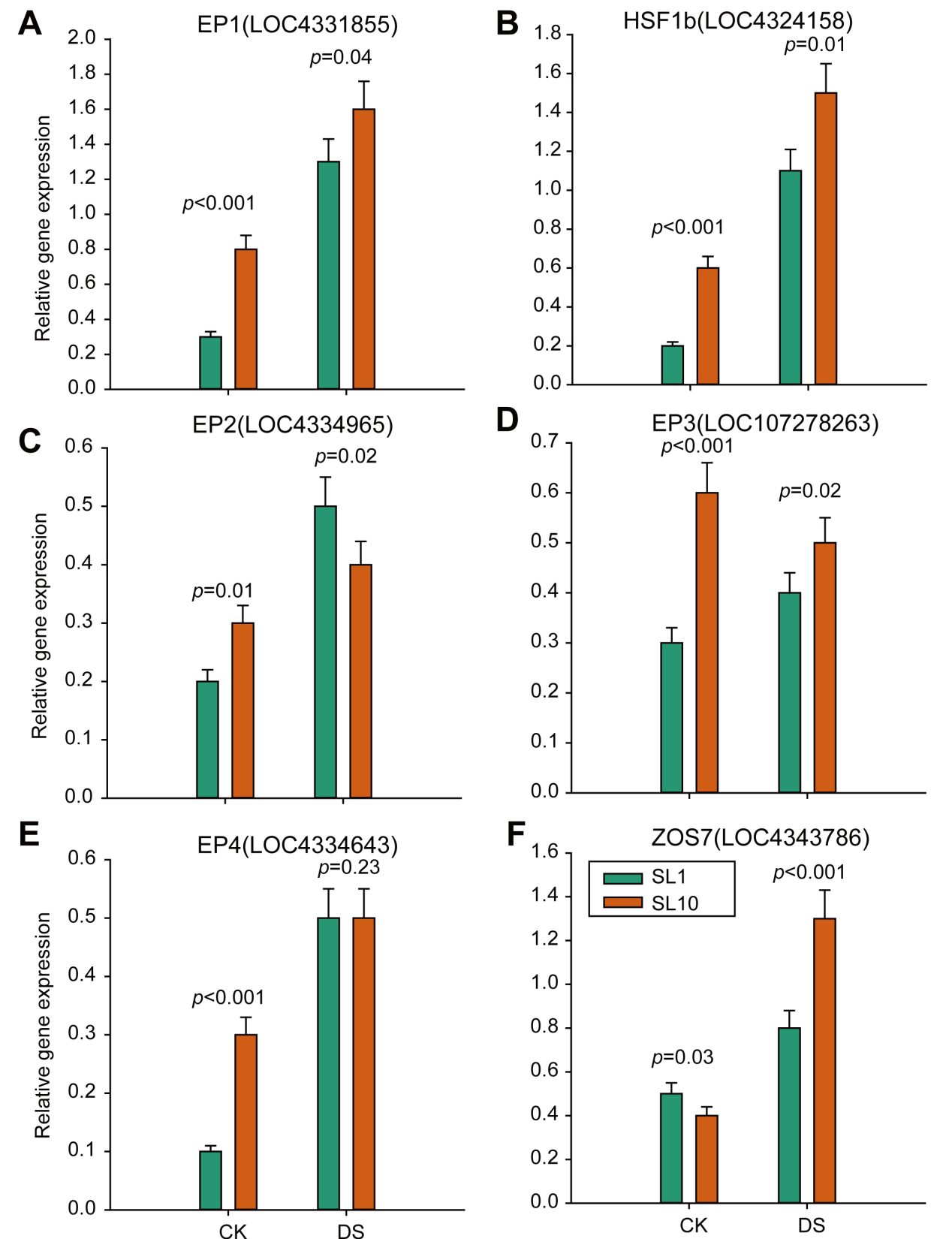


**Figure S8.** qPCR validation of DRGs in two Shanlan upland rice lines induced by drought stress. **A-E**, EP1(LOC4331855), HSF1b(LOC4324158), EP2(LOC4334965), EP3(LOC107278263), EP4(LOC4334643) and ZOS7 (LOC4343786). *P* values in each comparison were determined based on student *t*-test, respectively. *n*=3 (biological replicates).
